# Dental Society of the State of Maryland

**Published:** 1868-07

**Authors:** J. B. Bean, S. M. Field

**Affiliations:** Vice Pres. and Pres. Pro-tem.; Rec. Sec.


### CORRESPONDENCE.

### ARTICLE VII.

### *Dental Society of the State of Maryland.*

This association held its regular monthly meeting, Thursday evening, April 30th, at its rooms, in the Marble Building S. W. Corner of Lexington and Charles Streets.

After the usual formal business of the Society was transacted, the President stated that the propositions of Dr. Arthur, submitted at the January meeting and published in the March number of the "Journal" were next in order.* The truth of the position taken by Dr. A., and stated in these propositions, Dr. A. J. Volck undertook to deny.

Dr. Volck considered it incumbent upon Dr. Arthur in accordance with the usages of all scientific bodies to open the discussion by defending the propositions he had offered to the Society, and to state the facts and reasonings upon which he had based them.

Dr. Arthur replied that he had already presented his views

*1.—That caries will attack the proximate surfaces of all the teeth, except the inferior incisors, of the great majority of persons in the United States, at the present day.

When caries of the superior incisors occurs on the proximate surfaces previously to the twelfth year, its occurrence, sooner or later, on the same surfaces of all the teeth, except the inferior incisors is almost certain. In the greater number of such cases the caries will show itself before the twentieth year.

This predisposition to dental caries is greater in the female sex.

2d.—That caries is not liable to occur, at the points indicated, unless the teeth are in contact.

3d.—That an artificial, permanent separation of the teeth will arrest superficial caries or prevent its occurrence if the attack has not actually begun.

4th.—That it is popular fallacy to suppose that caries necessarily follows the removal of the enamel.

5th.—That the most efficient means of preserving the teeth is to anticipate the attack of caries by separating them, when it is ascertained that caries is likely to occur on the proximate surfaces.

in full detail to the association, and did not consider it at all necessary to go over the ground he had already traversed. He had not only presented the matter fully to the association, but had published his views. His little book had been ably and elaborately revised in the "*Am. Jour. of Dent. Science,*" by Prof: Noel. The whole subject, indeed, had been before the profession for some two years and, during that time, although Dr. A. had heard of a good deal said, privately, to patients who were entirely ignorant of the matter and could be misled to any extent by merely appealing to their crude and prejudiced notions, he had not become aware of any formal attempt having been made to disprove his views. That the gentleman had become acquainted with Dr. A's views, so publicly set forth, he must take for granted, as he could not, of course undertake to controvert the opinions and conclusion refered to, without first having made himself acquainted with them. As Dr. A. had not yet encountered any public opposition to his views, he had waited for the statement of the gentleman's ground of objection, and as he had understood he had prepared himself for the purpose. Dr. A. had come this evening, not to repeat views already presented to the Society but, to endeavor to defend those views from grounds of objection which had not yet presented themselves to his mind.

Dr. Volck said that he was aware that Dr. A. had published a pamphlet on his views of the treatment of dental caries, and had himself read both the pamphlet aud the review of Prof. Noel. That no reply had been made to Dr. A.'s published views, might be explained by the fact, that but few of the members of the dental profession were accustomed to writing and those who were found themselves generally too much occupied to give much time to the purpose. He did not doubt that Prof. N.'s review of Dr. A.'s pamphlet was particularly gratifying to Dr. A., but although Prof. N. was capable of writing well he had undertaken a subject in this case with which he was not acquainted. Dr. A.'s pamphlet was nothing more than the statement of the opinion of one man, and while he conceded the entire hon esty of intention of Dr. A., his mere personal opinion was not entitled to more credit than it was worth.

Dr. V. said he opposed the removal of the enamel of the teeth because he believed it to be furnished by nature as a protection from attacks of caries.

He had taken pains to examine, microscopically, sections of the teeth of a number of animals, (although he had not been able to bring his specimens with him,) and had found that, in the human teeth, the enamel was thickest in proportion to their size, of any he had examined. He concluded, from this fact, that nature had provided the human teeth with this additional protection to secure them from the attacks of caries to which they were subject.

He condemned filing sound teeth for the prevention of caries.

Dr. Arthur said he had come to the present meeting of the association, quite curious to ascertain what line of argument would be pursued by the gentlemen who had undertaken to dispute the truth of the views he had presented. He was gratified when Dr. V. announced his intention of undertaking to do this as he was recognized as being a man of intelligence and ability. As Dr. A. had no desire whatever, to hold any views of practice which could be shown to be erroneous, he was as much interested as any one, to discover errors which he might have adopted, He had, however, listened to what the gentleman had said but found in it not a shadow of argument. The comparative anatomy of the teeth, as referred to by Dr. V. had no bearing upon the subject. If the human teeth were covered with enamel an inch thick it would not alter the well known fact that caries of the teeth does commonly occur, at points where the enamel is most perfectly formed. The book, referred to, in the discussion. is not by any means, a mere record of the opinions of the writer. The views presented in it are merely the conclusions drawn from the observations of many years, and are all based upon facts. With regard to the removal of caries, without filling, for the arrest of this disease. Dr. A. stated that out of 1375 cases of removal of caries, failures had occurred in less than one hundred. In all these cases the enamel had been entirely removed from the affected surfaces, this is a fact.

Dr. A. declared it to be a mistake to suppose he had advocated the indiscriminate filing of sound teeth. He had done no such thing. The whole gist of the views he has so earnestly advocated is the anticipation of the attack of caries by separation in cases where it is well ascertained that it is certain to occur, and he endeavored to point out clearly the indications of a certain condition in which this treatment is advisable.

But it was useless, Dr. A. said, to waste further time or words on this discussion, in the form it had taken, and he should not do so, but when any objections to his views, worthy of attention, were offered, he would hold himself ready to give them his earnest consideration.

Dr. Bean desired, (as the subject was truly open to discussion,) to express his views on one or two points. He regarded Dr. Arthur's experience and his deductions therefrom, as of immense value to the profession and the community at large. Filling teeth for the removal or prevention of decay was not a new idea, or a new method of practice, but the *rule* which Dr. A's careful observation has established in regard to *the indications which foreshadow the certain decay of all the teeth on their approximal surfaces*, had been eminently verified in his (Dr. B's) own practice. The only exception to Dr. A's rule which had suggested itself, was in the case of young girls who have been in ill health during childhood, but, as is often the case, have acquired excellent health as they approached the period of mature womanhood. In such cases, we may find the superior incisors decayed at thirteen, but at sixteen, under improved constitutional conditions, we may find the causes of decay so far removed as to permanently arrest farther attack on the approximal surfaces of the bicuspids and molars. He thought Dr. A's exception of one in twenty might possibly provide for these cases.

Dr. Bean did not think the enamel so essential for the protection of the teeth from decay or chemical action, as from abrasion by grinding the food, and for this reason the enamel is always thickest on the cusps of the crowns in most animals, and really thinest on the approximal surfaces, and near the gums,where the teeth are most exposed to the agents of decay. The much larger per cent. of lime contained in the enamel, would argue that it would be more readily dissolved by acids. He did not believe that *a smooth polished surface of sound dentine* would be any more liable to attack by chemical agents which cause decay, than a similar surface of *enamel* under the same conditions. Decay is sometimes produced, and is often augmented by vitiated secretions from the stomach and from a diseased mucous membrane, but never from saliva itself; which is really the alkaline corrective provided by nature for these conditions. The almost universal agents of decay were most certainly those organic acids manufactured from the food and buccal secretions, by the various kinds of fermentation carried on in the mouth. In the localities indicated by Dr. Arthur between the teeth when in contact, and in cavities and crevices where these matters are protected from lavement by the saliva and the abrasion of mastication, all the elements for producing these various fermentations are abundant. In these undisturbed laboratories are produced successively renewed portions of *acetic*, *lactic* and *butyric* acids, which combine with the lime of the enamel and dentine, and at the same time continually enlarge the capacity of these localities for a continuously increasing supply of these corrosive agents.

J. B. Bean, D. D. S., *Vice Pres. and Pres. Pro-tem.*

S. M. Field, D. D. S., *Rec. Sec.*

---

